# NapR, a novel nucleoid-associated protein, regulates antioxidant defense in mycobacteria

**DOI:** 10.1128/msphere.00746-25

**Published:** 2025-11-13

**Authors:** Kun Wang, Xujie Cui, Xiangyang Zhang, Jiachen Zheng, Xiaocui Ling, Yunfan Zhang, Pengbo Yu, Boyan Lv, Weihui Li

**Affiliations:** 1 College of Life Science and Technology, Guangxi University, Guangxi Research Center for Microbial and Enzyme Engineering Technology, State Key Laboratory for Conservation and Utilization of Subtropical Agro-bioresources 622308, Nanning, China; The University of Iowa, Iowa City, Iowa, USA

**Keywords:** mycobacteria, antioxidant defense, transcriptional regulation, geranylgeranyl reductase

## Abstract

Nucleoid-associated proteins (NAPs) are essential in bacteria for maintaining nucleoid architecture and regulating the expression of target genes. Although some NAPs have been well studied in certain bacterial species, their specific functions and regulatory mechanisms remain poorly characterized in mycobacteria. In this study, we identified NapR as a novel nucleoid-associated protein in mycobacteria. We showed that NapR, which is highly conserved among mycobacteria, binds DNA and modulates DNA topology through bridging. Furthermore, we demonstrated that NapR acts as a positive transcriptional regulator of the *ggr* gene, which encodes geranylgeranyl reductase, thus regulating bacterial antioxidant defense. By controlling *ggr* expression, NapR modulates the level of intracellular ROS and influences the antioxidant capacity of mycobacteria. This study identifies NapR as a novel nucleoid-associated protein and defines a specific regulatory pathway involved in mycobacterial antioxidant defense, providing new insights into the mechanisms of the bacterial oxidative stress response.

**IMPORTANCE:**

As important global regulatory factors, nucleoid-associated proteins (NAPs) can help bacteria adapt to environmental stress, such as oxidative stress. However, the regulatory mechanism of NAPs in mycobacterial antioxidant defense is largely unclear and remains to be explored. Here, we identify NapR as a novel nucleoid-associated protein that modulates DNA topology by bridging. We revealed the regulatory effect of NapR on mycobacterial antioxidant defense. NapR positively regulates the expression of the geranylgeranyl reductase-encoding gene *ggr*. In addition, the ability of NapR to regulate the levels of intracellular ROS relies on *ggr*, ultimately leading to the antioxidant defense of *Mycobacterium smegmatis*. Our findings identify a new member of the NAP family and contribute to understanding the mechanisms of bacterial antioxidant defense.

## INTRODUCTION

Tuberculosis remains among the most widespread infectious diseases worldwide and is associated with high rates of morbidity and mortality in humans (1). Its pathogenic bacterium, *Mycobacterium tuberculosis*, is an intracellular pathogen that faces severe oxidative stress after being phagocytosed by macrophages. To counter oxidative stress, bacteria have evolved a series of antioxidant systems to increase the expression of antioxidant enzymes (2–4). The most basic method for countering oxidative stress in bacteria is to eliminate intracellular reactive oxygen species (ROS) through the activity of antioxidant enzymes such as superoxide dismutase, catalase, and peroxidase (5). In addition, transcriptional regulators can directly respond to oxidative signals and control bacterial antioxidant stress by regulating the expression of related genes. To date, several transcriptional regulators, such as OxyR and PerR, directly respond to oxidative stress and participate in regulating bacterial antioxidation (6). Nucleoid-associated proteins (NAPs) are also transcriptional regulatory proteins, but their regulation of bacterial antioxidative ability is still unclear, and the underlying mechanism remains to be explored.

NAPs are generally referred to as DNA-binding proteins and usually interact with transcriptional regulators to modulate the expression of target genes (7). Generally, NAPs can bend DNA by binding to the genome specifically or nonspecifically and induce topological and structural changes in chromosomal DNA, ensuring that they appropriately compress bacterial chromosomes (8). In addition, NAPs condense bacterial chromosomes by bridging and wrapping DNA to protect the DNA and maintain the integrity of the bacterial genome (9). Reportedly, most nucleoid-binding proteins exhibit relatively low binding sequence specificity, but their binding sites are usually rich in A and T bases (10, 11). In addition to their structural effects on the genome, nucleoid-binding proteins are involved in cellular processes such as transcription, DNA replication, DNA recombination, repair, and the SOS response (12–14). Research on NAPs in *Escherichia coli* started earlier than in other species and has been relatively extensive. At least 12 NAPs have been found in *E. coli*, such as heat-unstable nucleoid proteins, inversion stimulation factors, integration host factors, and chromosome structure maintenance proteins (15, 16). However, research on NAPs in mycobacteria is relatively recent. Like most gram-positive bacteria, only a few NAPs, such as Lsr2, mIHF, and NapM, have been found in mycobacteria (17–19).

Geranylgeranyl reductase (GGR) is encoded by *ggr* and has different functions across different species. GGR plays a crucial role in the biosynthesis of chlorophyll, including the reduction of geranylgeranyl pyrophosphate (GGPP) to phytyl pyrophosphate and the reduction of geranylgeranyl chlorophyll to chlorophyll, which is used by plants to produce energy through photosynthesis (20). In mycobacteria, GGR family proteins can catalyze the reduction of a single double bond in the side chain of mycobacterial menaquinone isoprene, accompanied by the dehydrogenation of FADH_2_, which is the redox regulation sensor of bacteria (21, 22). These results suggest that GGR decreases the reducing power and may be involved in bacterial antioxidant defense.

In this study, we report a novel nucleoid-associated protein, NapR, that negatively regulates mycobacterial antioxidant defense by positively regulating the expression of *ggr*. NapR positively mediates the levels of ROS depending on *ggr* in mycobacteria. Thus, this study expands the scope of NAPs identified in mycobacteria and establishes a link between NapR and mycobacterial antioxidant defense.

## RESULTS

### NapR negatively regulates the antioxidant activity of *Mycobacterium smegmatis*

To identify genes associated with oxidative stress, we screened a library of *M. smegmatis* transposon insertion mutants *in vitro* using H_2_O_2_ to mimic host infection conditions. Phenotypic rescreening revealed that a mutant strain (designated Msm/Tn) exhibited significantly enhanced resistance to 0.5 mM H_2_O_2_, whereas the growth of the wild-type strain (Msm/WT) was completely inhibited (Fig. S1). Sequencing revealed the insertion site within the *napR* gene, suggesting that NapR may negatively affect the antioxidant activity of *M. smegmatis*.

**Fig 1:**
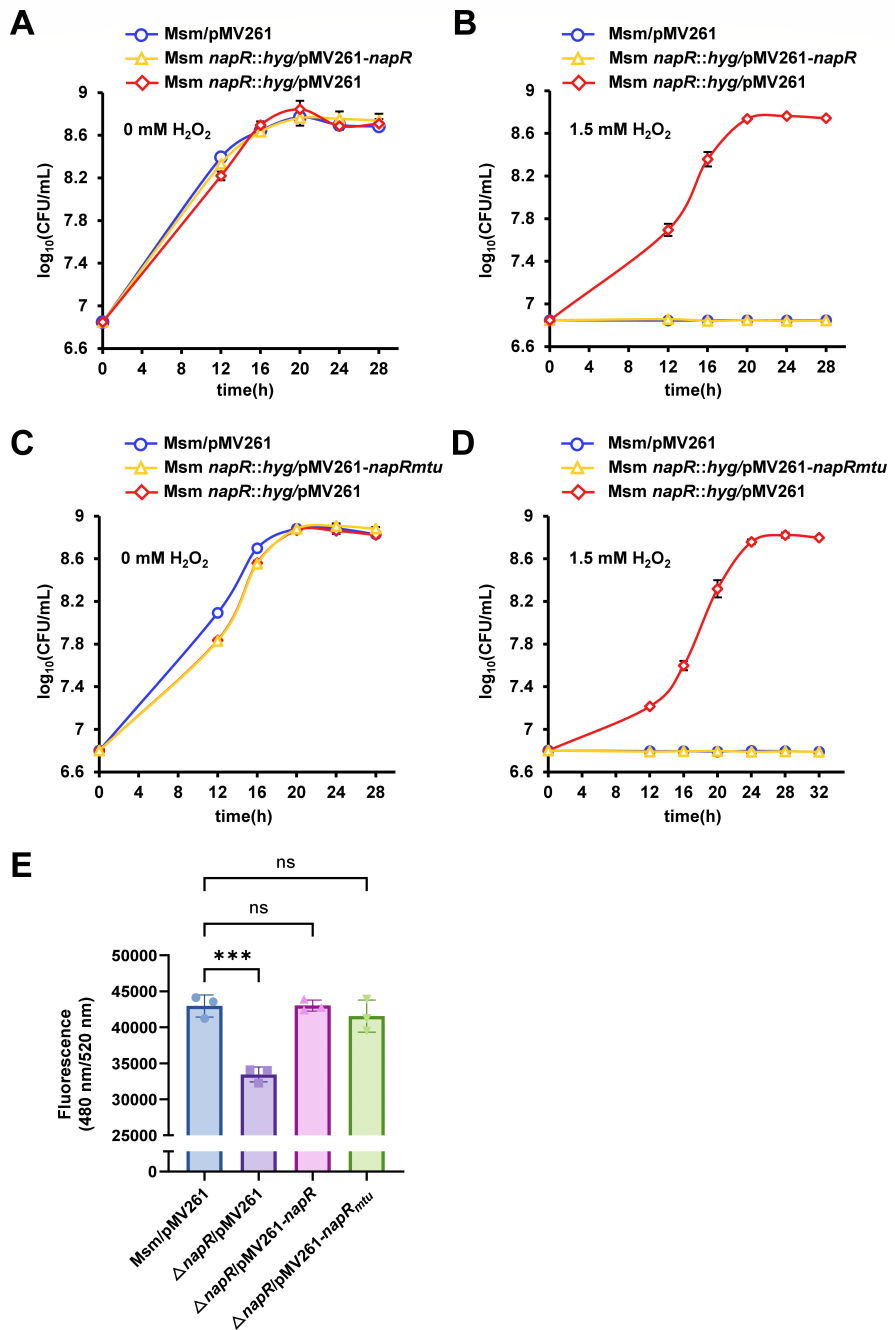
NapR negatively regulates the antioxidant growth of *M. smegmatis*. (**A**) Growth curves of the *M. smegmatis* wild-type strain (Msm/pMV261), *napR* deletion strain (Msm *napR::hyg*/pMV261), and complementary strain (Msm *napR::hyg*/pMV261-*napR*) without H_2_O_2_. (**B**) Growth curves of the *M. smegmatis* wild-type strain (Msm/pMV261), *napR* deletion strain (Msm *napR::hyg*/pMV261), and complementary strain (Msm *napR::hyg*/pMV261-*napR*) with 1.5 mM H_2_O_2_. (**C**) Growth curves of the *M. smegmatis* wild-type strain (Msm/pMV261), *napR*-deleted strain (Msm *napR::hyg*/pMV261), and cross-complementary strain (Msm *napR::hyg*/pMV261-*napR_mtu_*) without H_2_O_2_. (**D**) Growth curves of the *M. smegmatis* wild-type strain (Msm/pMV261), *napR*-deleted strain (Msm *napR::hyg*/pMV261), and cross-complementary strain (Msm *napR::hyg*/pMV261-*napR_mtu_*) with 1.5 mM H_2_O_2_. (**E**) Relative ROS levels of the *M. smegmatis* wild-type strain (Msm/pMV261), *napR*-deleted strain (Msm *napR::hyg*/pMV261), complementary strain (Msm *napR::hyg*/pMV261-*napR*), and cross-complementary strain (Msm *napR::hyg*/pMV261-*napR_mtu_*). The values presented are the averages of three independent experiments. Three asterisks (***) in the figure denote a significant difference (*P* < 0.001, two-tailed Student’s *t*-test) between two groups. ns, not significant. The error bars represent the standard deviation of the data derived from three biological replicates.

To confirm the role of NapR in antioxidant defense, we constructed *napR* deletion mutant and complementation strains in *M. smegmatis*. As shown in Fig. 1A and Fig. S4A, in the absence of H_2_O_2_, there was no difference in growth between the recombinant strains (wild-type strain, *napR*-deleted strain, and complementary strain) in 7H9 medium. However, when exposed to 1.5 mM H_2_O_2_, the *napR*-deleted strain (Msm *napR::hyg*/pMV261) exhibited robust growth, whereas both the wild-type strain (Msm/pMV261) and the complementation strain (Msm *napR::hyg*/pMV261-*napR*) showed significant growth inhibition (Fig. 1B and Fig. S4A). These results demonstrate that NapR negatively regulates the antioxidant growth of *M. smegmatis*.

Next, we explored the conservation of NapR in several common mycobacteria. The results revealed 76.6% amino acid sequence identity in NapR between *M. smegmatis* and *M. tuberculosis*, while the NapR amino acid sequences in *M. tuberculosis* H37Rv, *M. tuberculosis* H37Ra, and *Mycobacterium bovis* BCG were completely identical (Fig. S2). We investigated whether the ortholog of NapR in *M. tuberculosis* shares this function. With respect to the heterologous expression of Rv3050c (NapR_mtu_) in the *M. smegmatis napR*-deleted strain, the sensitivity of the *napR*-deleted strain to H_2_O_2_ was restored to a level comparable to that of the wild-type strain (Fig. 1D and Fig. S4B), while there were no significant differences in growth under nonstress conditions (Fig. 1C and Fig. S4B). These results suggest that *M. tuberculosis* NapR_mtu_ and *M. smegmatis* NapR play a conserved role in regulating antioxidant defense.

ROS play essential roles in bacterial antioxidant defense. We measured the levels of ROS in the following strains of *Mycobacterium smegmatis*: the wild-type strain (Msm/pMV261), the *napR*-deleted strain (Msm *napR::hyg*/pMV261), a complementary strain (Msm *napR::hyg*/pMV261-*napR*), and the cross-complementary strain (Msm *napR::hyg*/pMV261-*napR_mtu_*). As shown in Fig. 1E, the ROS levels in the *napR*-deleted strain were significantly lower than those in the wild-type strain. Both the complementary strain and the cross-complementary strain restored the ROS levels to the wild-type levels. These data indicate that NapR positively regulates the ROS levels in *M. smegmatis*, thus affecting bacterial antioxidant defense.

### NapR is a novel nucleoid-associated protein in *M. smegmatis*

**Fig 2:**
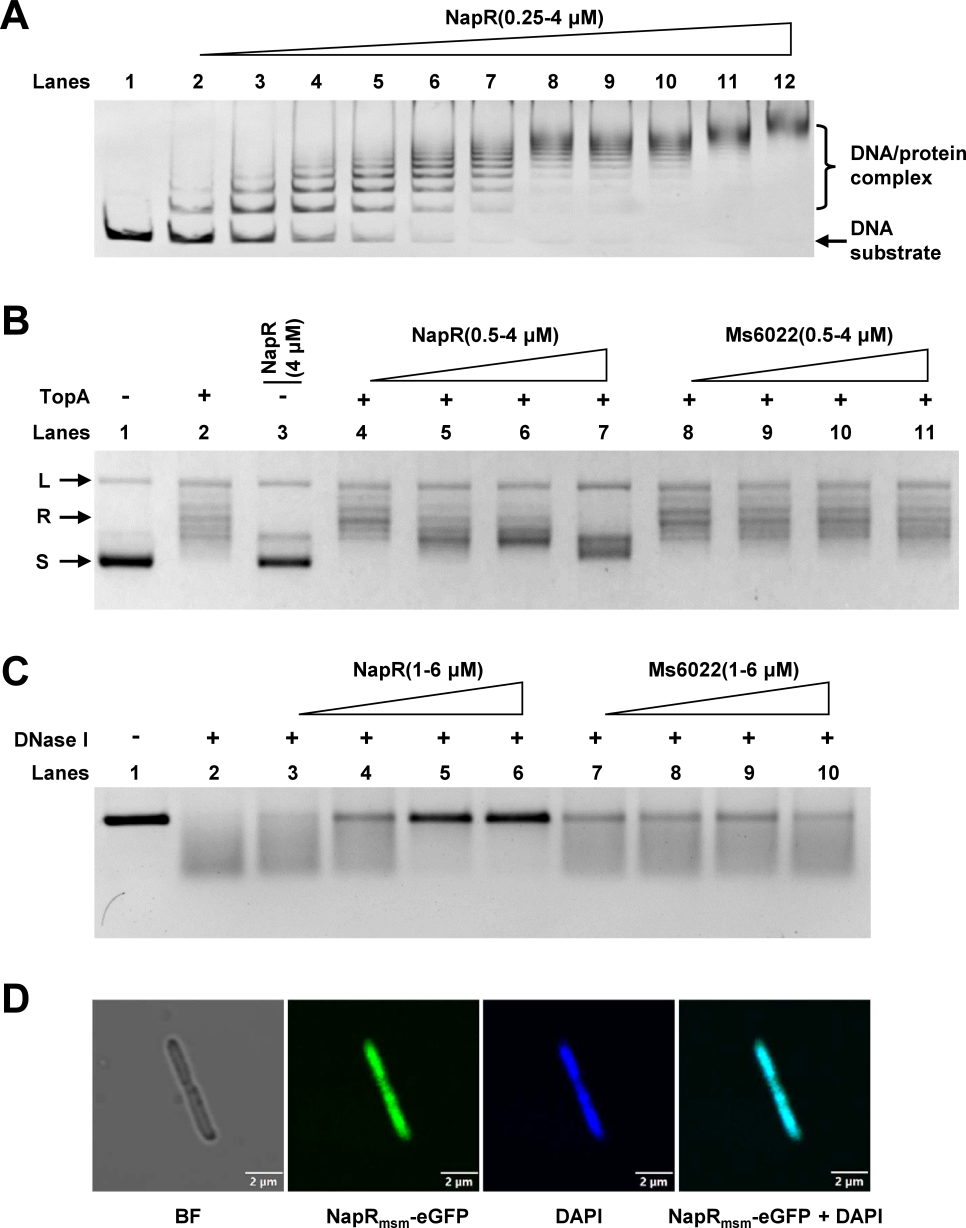
*M. smegmatis* NapR is a novel nucleoid-associated protein. (**A**) EMSA of NapR. EMSAs of the binding of NapR to its own promoter. In lanes 1–12, the concentrations of NapR were 0, 0.25, 0.5, 0.75, 1, 1.25, 1.5, 1.75, 2, 2.5, 3, and 4 µM, respectively. (**B**) Topoisomerase unwinding experiments. Lane 1 represents the supercoiled plasmid. Lane 2 is the plasmid conformation relaxed by Msm TopA. Lane 3 aims to verify that NapR has no conformational effect on supercoiled plasmids. Lanes 4–7 show the results of the addition of TopA after the supercoiled plasmid was incubated with 0.5, 1, 2, and 4 µM NapR, respectively. Lanes 8–11 show the negative control groups. (**C**) DNase I digestion experiment. Lane 1 represents the linearized DNA. Lane 2 represents the DNA completely degraded by DNase I. Lanes 3–6 show the results of the addition of DNase I after the linear DNA was incubated with 1, 2, 4, and 6 µM NapR, respectively. Lanes 7–10 show the negative control groups. (**D**) Colocalization assays for enhanced green fluorescent protein (eGFP)-tagged NapR with the nucleoid in *M. smegmatis*. From left to right: BF, bright-field observation of *M. smegmatis* morphology; NapR-eGFP, NapR was visualized by fusing with eGFP; DAPI, nucleoid was detected by staining with DAPI; NapR-eGFP + DAPI, superimposition of eGFP-tagged NapR and DAPI-stained nucleoid. The scale bar in each figure represents 2 µm.

NapR is annotated as a transcriptional regulator in *M. smegmatis*. To explore its transcriptional regulatory function, we expressed and purified NapR and then investigated its DNA-binding activity via an electrophoretic mobility shift assay (EMSA). We found that NapR could bind to its own promoter, resulting in the formation of multiple hysteresis bands of protein–DNA complexes, and as the concentration of NapR increased, the number of hysteresis gel bands gradually increased (Fig. 2A). This finding is consistent with the properties of nucleoid-associated proteins. Therefore, we speculated that NapR might be a nucleoid-associated protein.

The term “nucleoid-associated protein” indicates that it nonspecifically binds to DNA and protects the conformation of DNA against being changed. To verify this, we first evaluated the effect of NapR on the unwinding activity of TopA (topoisomerase) *in vitro*. As shown in Fig. 2B, TopA unwound the supercoiled plasmids (lane 2), whereas NapR had no effect on the conformation of the supercoiled plasmids (lane 3). However, with increasing concentrations of NapR, the quality of protein-binding plasmids unwound by TopA decreased (lanes 3–7), as the wrapping of NapR around the plasmid prevented TopA from unwinding it. In contrast, under similar conditions, the control transcription factor MSMEG_6022 had no effect on the plasmid unwound by TopA (lanes 8–11). In conclusion, NapR is a nucleoid-associated protein that protects the supercoiled conformation of plasmids from unwinding by TopA.

Furthermore, we confirmed our hypothesis by performing DNase I digestion experiments to verify the protective effect of NapR on linear DNA fragments. DNase I digested virtually all of the linearized DNA fragments, resulting in undetectable complete DNA fragments in the samples without NapR (Fig. 2C, lane 2). However, with increasing concentrations of NapR in the reaction mixture, a clear DNA band was observed in the gel (lanes 3–6), indicating the wrapping effect of NapR on DNA. In contrast, MSMEG_6022, a control transcription factor, did not exhibit this ability under similar conditions (lanes 7–10). Therefore, NapR is a nucleoid-associated protein that protects DNA from DNase I digestion.

Owing to the nonspecific binding of DNA, nucleoid-associated proteins are consistent with chromatin DNA in terms of their intracellular localization. To investigate whether NapR also has this characteristic, we conducted fluorescence colocalization experiments. We fused NapR and enhanced green fluorescent protein (eGFP) to locate NapR through fluorescence detection. The bacterial chromatin DNA was stained and located using the fluorescent dye DAPI. As shown in Fig. 2D, cyan fluorescence was viewed by confocal fluorescence microscopy because of the colocalization of the green fluorescent NapR-eGFP fusion protein and the blue fluorescent chromatin DNA. These results suggest that NapR can bind to chromatin DNA *in vivo*, further suggesting that NapR is a nucleoid-associated protein.

In summary, collective experimental evidence confirmed that NapR is a nucleoid-associated protein. These results indicate that NapR is a novel nucleoid-associated protein in *M. smegmatis*.

### NapR_mtu_ is a novel nucleoid-associated protein in *M. tuberculosis*

**Fig 3:**
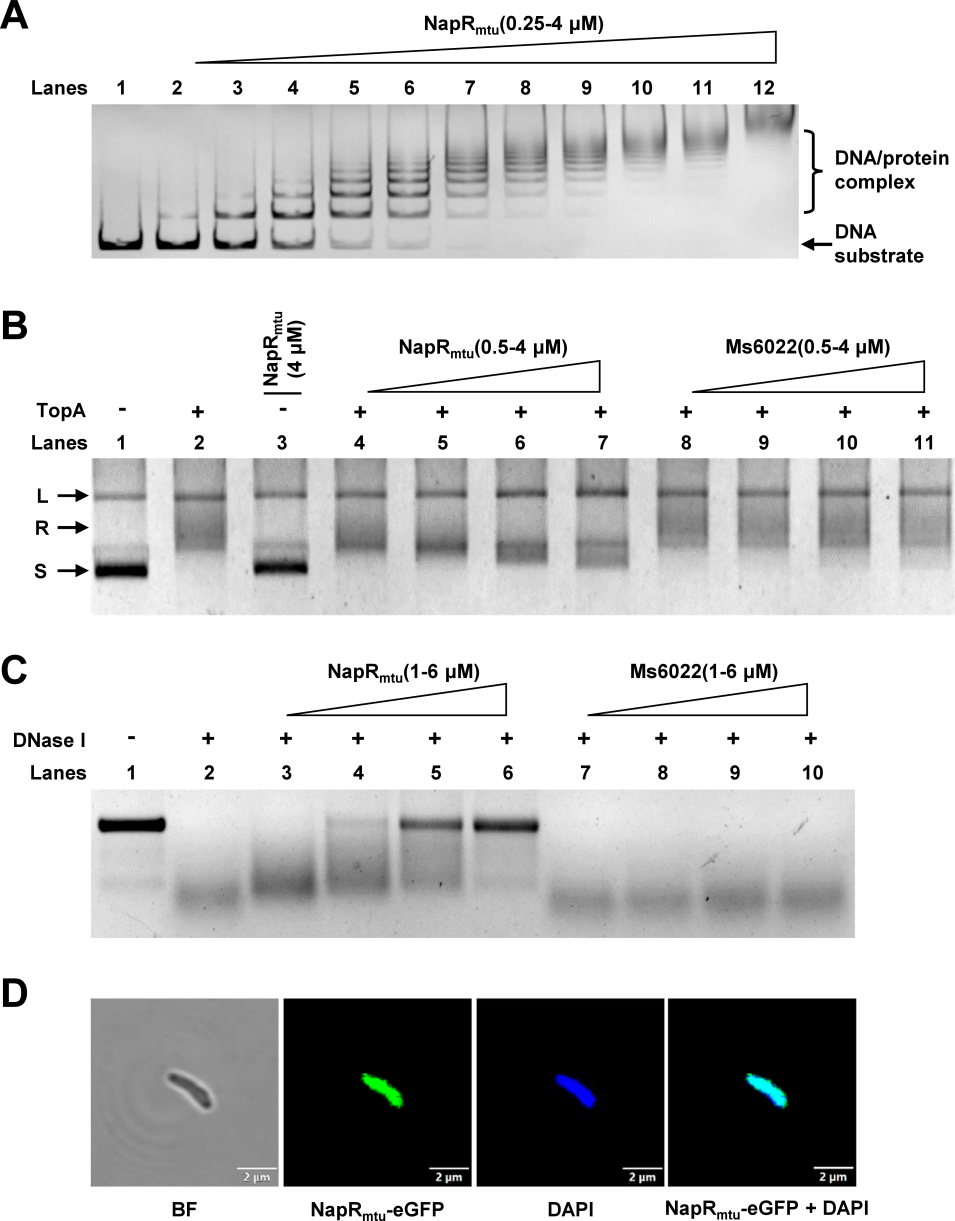
*M. tuberculosis* NapR_mtu_ is a novel nucleoid-associated protein. (**A**) EMSA of NapR_mtu_. EMSAs of NapR_mtu_ binding to its own promoter. In lanes 1–12, the concentrations of NapR_mtu_ were 0, 0.25, 0.5, 0.75, 1, 1.25, 1.5, 1.75, 2, 2.5, 3, and 4 µM, respectively. (**B**) Topoisomerase unwinding experiments. Lane 1 represents the supercoiled plasmid. Lane 2 represents the plasmid conformation relaxed by Msm TopA. Lane 3 aims to verify that NapR_mtu_ has no conformational effect on supercoiled plasmids. Lanes 4–7 show the results of the addition of TopA after the supercoiled plasmid was incubated with 0.5, 1, 2, and 4 µM NapR_mtu_, respectively. Lanes 8–11 show the negative control groups. (**C**) DNase I digestion experiment. Lane 1 represents the linearized DNA. Lane 2 represents the DNA completely degraded by DNase I. Lanes 3–6 represent the results of the addition of DNase I after the linear DNA was incubated with 1, 2, 4, and 6 µM NapR_mtu_, respectively. Lanes 7–10 show the negative control groups. (**D**) Colocalization assays for eGFP-tagged NapR_mtu_ with the nucleoid in *M. tuberculosis* H37Ra. From left to right: BF, bright-field observation of *M. tuberculosis* morphology; NapR_mtu_-eGFP, NapR_mtu_ was visualized by fusing with eGFP; DAPI, a nucleoid was detected by staining with DAPI; and NapR_mtu_-eGFP + DAPI, superimposition of eGFP-tagged NapR_mtu_ and DAPI-stained nucleoids. The scale bar in each figure represents 2 µm.

The conservation of the protein amino acid sequence (Fig. S2) indicates that it may also be conserved in function. We therefore hypothesized that *M. tuberculosis* NapR_mtu_ may also be a nucleoid-associated protein. To verify this, we first analyzed the DNA-binding activity of NapR_mtu_. As shown in Fig. 3A, NapR_mtu_ binds to the promoter of its own gene, resulting in the formation of lagging gel bands of multiple protein–DNA complexes. Hence, it can be preliminarily implied that NapR_mtu_ might also be a nucleoid-associated protein.

Next, we evaluated the effect of NapR_mtu_ on the unwinding activity of TopA *in vitro*. As shown in Fig. 3B, the addition of NapR_mtu_ reduced the relaxation ability of TopA on the supercoiled plasmid (lanes 4–7), which was due to the wrapping of NapR_mtu_ around the plasmid. Subsequently, the degradation of linear DNA by DNase I was also weakened by NapR_mtu_ (Fig. 3C). In contrast, the corresponding control transcriptional regulator, MSMEG_6022, had no effect on the activity of TopA or DNase I. These results indicate that NapR_mtu_ can wrap supercoiled plasmids and linear DNA to protect them from enzymes, which is a typical characteristic of NAPs. Furthermore, as shown in Fig. 3D, NapR_mtu_ and chromatin DNA can be colocalized in *M. tuberculosis* H37Ra, indicating that NapR_mtu_ can bind to chromatin DNA. In addition, the same fluorescence colocalization experiments were performed in *M. bovis* BCG (Fig. S3).

In summary, NapR_mtu_, like NapR, also possesses the characteristics of a nucleoid-associated protein. These results suggest that NapR is a novel nucleoid-associated protein in mycobacteria.

### NapR modifies DNA topology through a bridging effect

**Fig 4:**
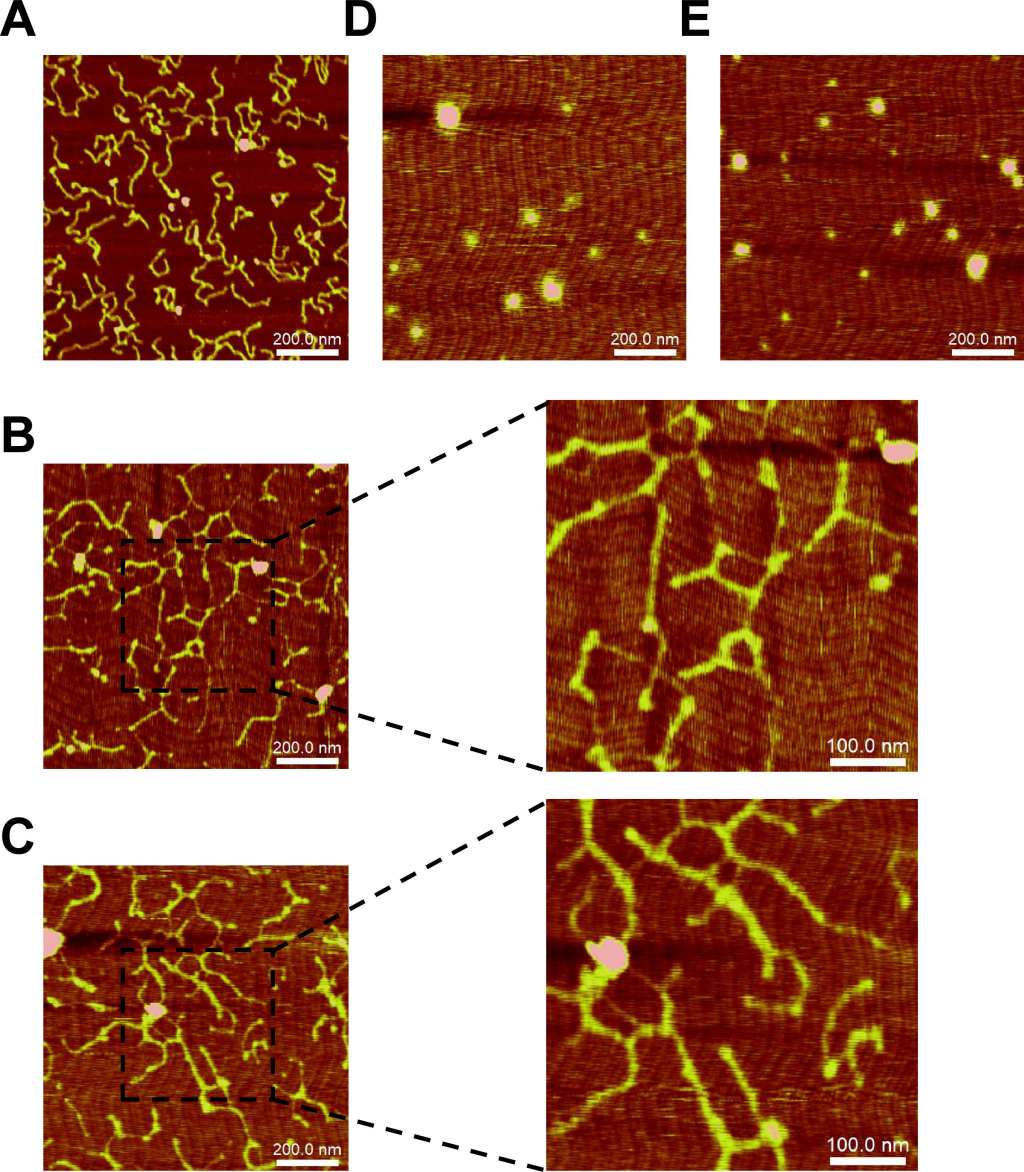
NapR is a DNA-topology modulator through bridging DNA. (**A**) Free DNA fragment in the absence of the NapR protein. (**B**) Images of the protein–DNA complexes formed by NapR_msm_ and DNA fragments at the protein:DNA ratios (monomer per 140 bp). (**C**) Images of the protein–DNA complexes formed by NapR_mtu_ and DNA fragments at the protein:DNA ratios (monomer per 140 bp). (**D**) Image of NapR_msm_ without DNA fragments. (**E**) Image of NapR_mtu_ without DNA fragments. The same color scale range was used for all the images.

To investigate the structural consequences of the binding of NapR to DNA and elucidate the underlying molecular mechanism, we employed atomic force microscopy (AFM) to directly visualize the NapR–DNA complexes at near-atomic resolution. As shown in Fig. 4A, the AFM images of the DNA-only control samples exhibited relaxed, extended conformations consistent with those of unbound DNA. In contrast, incubation of DNA with NapR_msm_ resulted in dramatic structural reorganization: DNA fragments were recruited and assembled into distinct bundle-like and mesh-like structures (Fig. 4B). Similarly, incubation with NapR_mtu_ also induced the formation of analogous higher-order structures (Fig. 4C). When imaged in isolation on mica, both NapR_msm_ and NapR_mtu_ appeared as spheroids without aggregation (Fig. 4D and E).

These results indicate that NapR acts as a DNA-bridging protein. NapR promotes extensive intermolecular interactions between DNA fragments, leading to their condensation into large complexes characterized by bundled and meshed architectures. This DNA-bridging activity provides a direct mechanistic basis for the ability of NapR to be modulated as a nucleoid-associated protein.

### NapR positively regulates the expression of GGR

**Fig 5:**
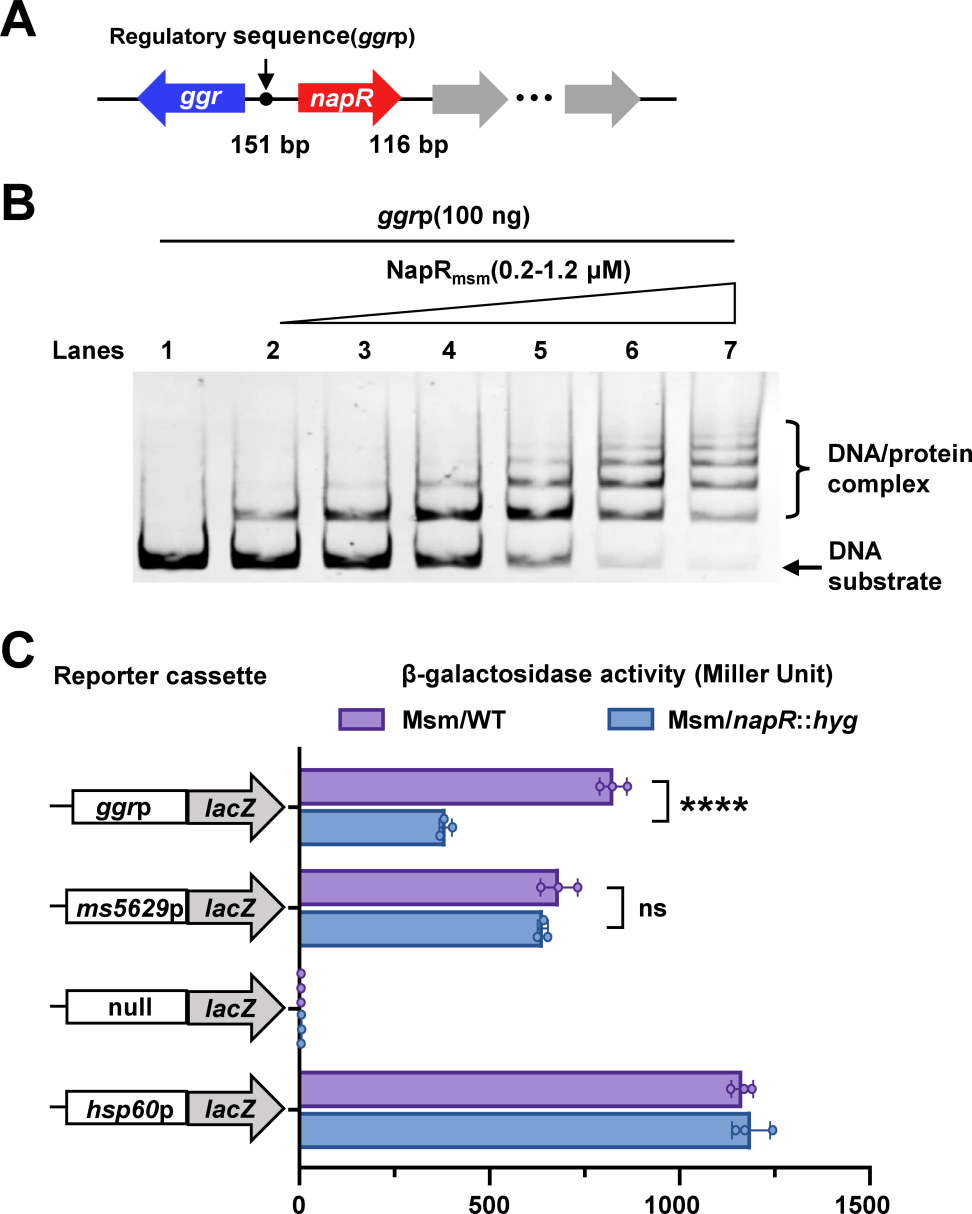
NapR positively regulates the expression of *ggr*. (**A**) Schematic of the *napR* operon and its regulatory region. (**B**) EMSA of NapR. In lanes 1–7, the concentrations of NapR were 0, 0.2, 0.4, 0.6, 0.8, 1, and 1.2 µM, respectively. (**C**) β-Galactosidase activity assays. The left side of the figure shows a schematic of the recombinant plasmid structure, and the right side of the figure shows the measured β-galactosidase activity (Miller units). The red bar represents the *M. smegmatis* wild-type strain (Msm/WT), and the blue bar represents the *napR*-deleted strain (Msm/*napR::hyg*). Null promoter-*lacZ*, *hsp60-lacZ,* and *ms5629*p-*lacZ* were used as controls. The values presented are the averages of three independent experiments. The four asterisks (****) in the figure denote a significant difference (*P* < 0.0001, two-tailed Student’s *t*-test) between two groups. ns, not significant. The error bars represent the standard deviation of the data derived from three biological replicates.

To explore downstream targets related to antioxidant activity regulated by NapR, we investigated the annotation information of genes adjacent to *napR* in the genome, focusing on the geranylgeranyl reductase-encoding gene (*ggr*), which is involved in redox processes (Fig. 5A). Next, we analyzed the binding of NapR and the *ggr* promoter by EMSA. As shown in Fig. 5B, NapR directly binds to the *ggr* promoter.

β-Galactosidase activity assays were subsequently performed to examine the specific regulatory effects of NapR on *ggr* expression using recombinant *lacZ* reporter plasmids. As shown in Fig. 5C, compared with the nonpromoter *lacZ* plasmid, *hsp60* strongly promoted the expression of *lacZ* in the wild-type and mutant strains of *M. smegmatis*, indicating that the reporting system worked well. No significant difference in *lacZ* activity was detected between the wild-type and *napR*-deleted strains when the *ms5629*p promoter was used as a control. However, compared with that of the wild-type strain, the *ggr*p-*lacZ* activity of the *napR* deletion strain was significantly lower. The significant reduction in *ggr*p-*lacZ* enzyme activity caused by *napR* deletion indicates that NapR acts as an activator and positively regulates the expression of *ggr*, a redox-related geranylgeranyl reductase-encoding gene.

### GGR affects the antioxidant defense of *M. smegmatis* by positively regulating the levels of ROS

**Fig 6:**
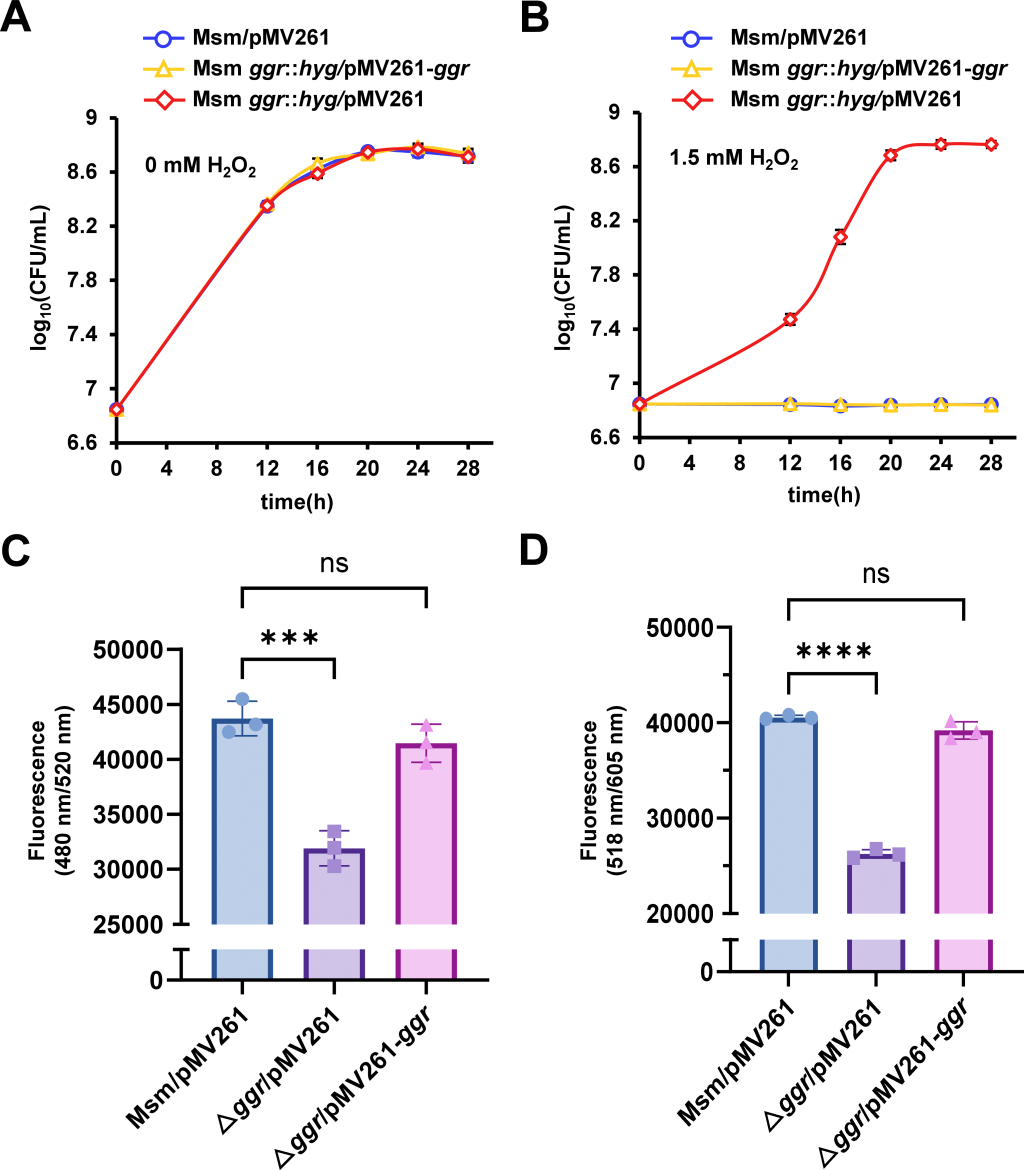
GGR affects the antioxidant defense of *M. smegmatis* by positively regulating the levels of ROS. (**A**) Growth curves of the *M. smegmatis* wild-type strain (Msm/pMV261), *ggr*-deleted strain (Msm *ggr::hyg*/pMV261), and complementary strain (Msm *ggr::hyg*/pMV261-*ggr*) without H_2_O_2_. (**B**) Growth curves of the *M. smegmatis* wild-type strain (Msm/pMV261), *ggr*-deleted strain (Msm *ggr::hyg*/pMV261), and complementary strain (Msm *ggr::hyg*/pMV261-*ggr*) with 1.5 mM H_2_O_2_. (**C**) Relative ROS levels of the *M. smegmatis* wild-type strain (Msm/pMV261), *ggr*-deleted strain (Msm *ggr::hyg*/pMV261), and complementary strain (Msm *ggr::hyg*/pMV261-*ggr*). (**D**) Relative superoxide anion (O_2_^-^) levels of the *M. smegmatis* wild-type strain (Msm/pMV261), *ggr*-deleted strain (Msm *ggr::hyg*/pMV261), and complementary strain (Msm *ggr::hyg*/pMV261-*ggr*). The values presented are the averages of three independent experiments. ****, *P* < 0.0001; ***, *P* < 0.001; ns, not significant (two-tailed Student’s *t*-test). The error bars represent the standard deviation of the data derived from three biological replicates.

GGR has been annotated as a redox-related geranylgeranyl reductase, which suggests that it may affect the efficiency of electron transport and thus be involved in mycobacterial antioxidant activity. To verify this, we constructed *ggr*-related recombinant strains and determined their growth curves. As shown in Fig. 6A and Fig. S4C, no significant differences were observed in the growth states of the three strains in the absence of H_2_O_2_. In contrast, the resistance of the wild-type strain (Msm/pMV261) and the complementary strain (Msm *ggr::hyg*/pMV261-*ggr*) was significantly inhibited under 1.5 mM H_2_O_2_ conditions, whereas the *ggr*-deleted strain (Msm *ggr::hyg*/pMV261) exhibited significant H_2_O_2_ resistance (Fig. 6B and Fig. S4C).

To investigate the mechanism by which GGR affects bacterial antioxidant defense, we measured the levels of ROS in the *M. smegmatis* wild-type strain (Msm/pMV261), *ggr*-deleted strain (Msm *ggr::hyg*/pMV261), and complementary strain (Msm *ggr::hyg*/pMV261-*ggr*). As shown in Fig. 6C, the levels of ROS in the *ggr*-deleted strain were significantly lower than those in the wild-type and complementary strains. The superoxide anion (O_2_^-^), a type of ROS, results from the reduction of O_2_ and has the capacity to damage components of the electron transport chain (23). We found that the levels of superoxide anions in the *ggr*-deleted strain were significantly lower than those in the wild-type and complementary strains (Fig. 6D). In summary, the deletion of *ggr* causes decreased levels of ROS, including superoxide anions.

These results indicate that GGR positively affects the levels of ROS in *M. smegmatis*, ultimately affecting bacterial antioxidant defense.

### NapR-mediated regulation of the antioxidant activity of *M. smegmatis* relies on *ggr*

**Fig 7:**
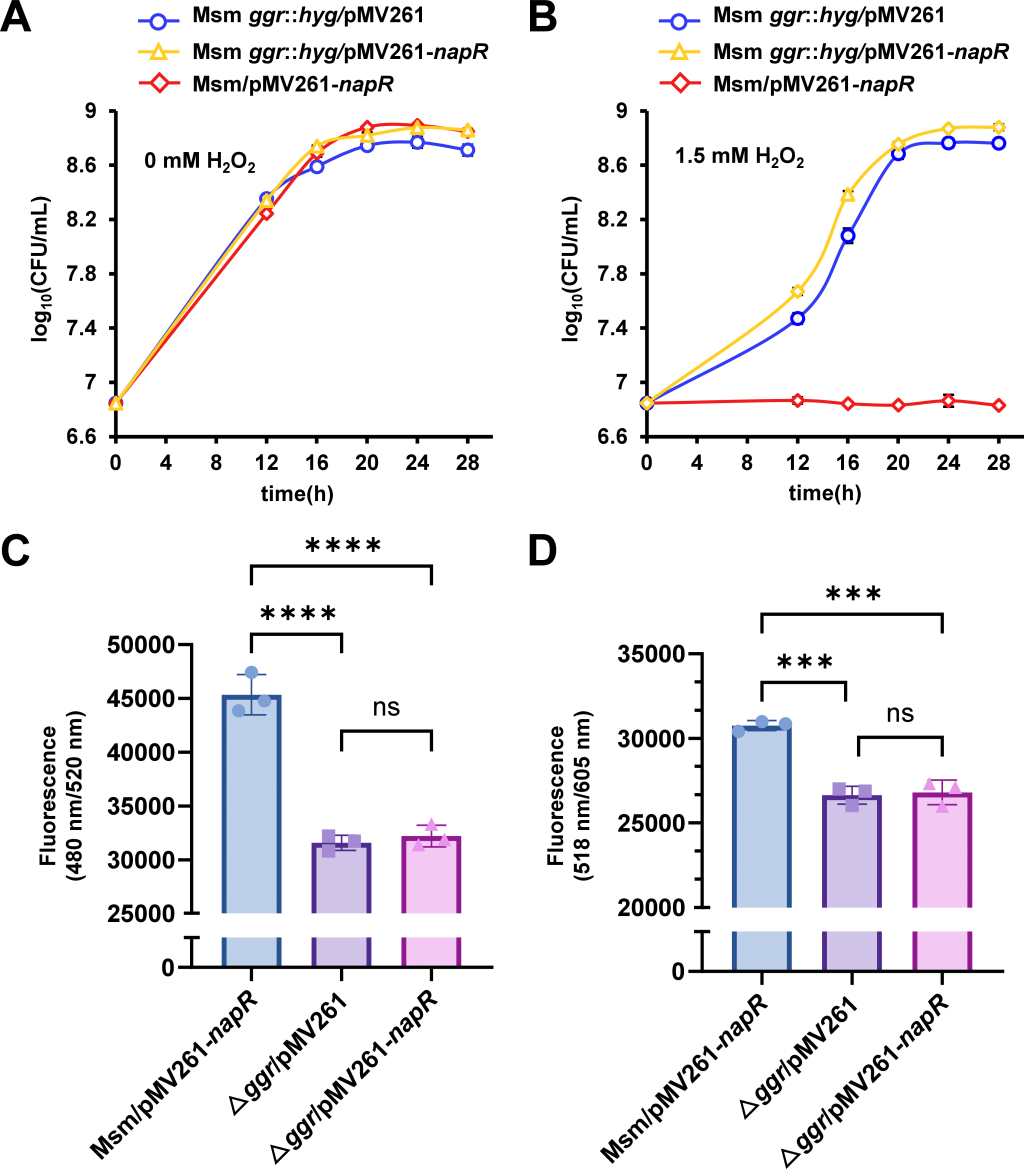
NapR regulates the antioxidant activity of *M. smegmatis* in a *ggr*-dependent manner. (**A**) Growth curves of the *napR*-overexpressing strain (Msm/pMV261-*napR*), *ggr*-deleted strain (Msm *ggr::hyg*/pMV261), and cross-recombinant strain (Msm *ggr::hyg*/pMV261-*napR*) without H_2_O_2_. (**B**) Growth curves of the *napR*-overexpressing strain (Msm/pMV261-*napR*), *ggr*-deleted strain (Msm *ggr::hyg*/pMV261), and cross-recombinant strain (Msm *ggr::hyg*/pMV261-*napR*) with 1.5 mM H_2_O_2_. (**C**) Relative ROS levels of the *M. smegmatis napR*-overexpressing strain (Msm/pMV261-*napR*), *ggr*-deleted strain (Msm *ggr::hyg*/pMV261), and cross-recombinant strain (Msm *ggr::hyg*/pMV261-*napR*). (**D**) Relative superoxide anion (O_2_^-^) levels of the *M. smegmatis napR*-overexpressing strain (Msm/pMV261-*napR*), *ggr*-deleted strain (Msm *ggr::hyg*/pMV261), and cross-recombinant strain (Msm *ggr::hyg*/pMV261-*napR*). The values presented are the averages of three independent experiments. ****, *P* < 0.0001; ***, *P* < 0.001; ns, not significant (two-tailed Student’s *t*-test). The error bars represent the standard deviation of the data derived from three biological replicates.

Our previous results revealed that NapR positively regulates the expression of *ggr* and negatively regulates the antioxidant defense of *M. smegmatis*. Furthermore, the above results demonstrate that GGR mediates the antioxidant activity of *M. smegmatis*. Therefore, we speculate that the ability of NapR to regulate the antioxidant activity of *M. smegmatis* relies on *ggr*. To confirm this speculation, we overexpressed the *napR* gene in the *ggr*-deleted and wild-type strains. As shown in Fig. 7A and Fig. S4D, there were no significant differences in the growth states of these strains in the absence of H_2_O_2_. In contrast, the *napR*-overexpressing strain (Msm/pMV261-*napR*) was significantly inhibited by 1.5 mM H_2_O_2_, whereas the cross-recombinant strain (Msm *ggr::hyg*/pMV261-*napR*) was significantly resistant to H_2_O_2_, which was similar to the *ggr*-deleted strain (Msm *ggr::hyg*/pMV261) (Fig. 7B and Fig. S4D). This finding indicates that the expression of *napR* in the *ggr*-deleted strain has no effect on its antioxidant activity because the deletion of *ggr* blocks the regulatory pathway of NapR. Meanwhile, we constitutively expressed *ggr* in the *napR* deletion strain background. The expression of *ggr* fully restored the wild-type level of resistance to H_2_O_2_ in the *napR* deletion strain (Fig. S5). Hence, our results show that the regulation of mycobacterial antioxidant defense by NapR depends on *ggr*.

On the basis of the above results, in conjunction with the previous evidence that GGR and ROS are positively correlated, we measured the levels of ROS and superoxide anion (O_2_^-^) in the *M. smegmatis napR*-overexpressing strain (Msm/pMV261-*napR*), *ggr*-deleted strain (Msm *ggr::hyg*/pMV261), and cross-recombinant strain (Msm *ggr::hyg*/pMV261-*napR*). As shown in Fig. 7C and D, the simultaneous expression of *napR* in strains with or without *ggr* resulted in significant differences in ROS and superoxide anion (O_2_^-^) levels, indicating the importance of GGR in maintaining ROS levels in *M. smegmatis*. Furthermore, there were no significant differences in the levels of ROS in *ggr*-deleted and cross-recombinant strains. The deletion of *ggr* results in NapR having no effect on the levels of ROS, as the regulation of ROS by NapR depends on *ggr*.

In brief, the antioxidant defense pathway regulated by NapR revealed that NapR positively regulates the expression of *ggr*, which in turn affects the levels of ROS in *M. smegmatis*, ultimately leading to an impact on bacterial antioxidant defense.

## DISCUSSION

NAPs play important roles in global regulation in bacteria. Therefore, the discovery of new NAPs is highly important for the complementation of bacterial regulatory networks. In this study, we verified that NapR is a novel nucleoid-associated protein that regulates mycobacterial antioxidant defenses through the expression of *ggr*, encoding geranylgeranyl reductase. Our findings add new members to the collection of known NAPs and suggest a link between the regulation of NapR and bacterial adaptation to antioxidant defense.

Various NAPs play a variety of roles in bacteria by condensing chromosomal DNA to modulate gene expression. Genome remodeling is driven by four key molecular mechanisms involving NAPs: (i) local DNA bending; (ii) global DNA wrapping; (iii) bridging of distal DNA segments; and (iv) nucleoprotein fiber assembly (24). We observed that, similar to Lsr2, NapR modulates DNA topology by bridging DNA segments (Fig. 3B and C). The *Mycobacterium tuberculosis* Lsr2 protein is considered the first identified H-NS analog in gram-positive bacteria (25). AFM analysis revealed that Lsr2 plays a distinct role in DNA bridging (26). H-NS is a conserved nucleoid-associated protein in bacteria that organizes chromatin via DNA bridging (27). Functionally, NapR parallels H-NS but has distinct physicochemical properties. Histone-like nucleoid structuring protein (H-NS) is a 15.4 kDa alkaline protein conserved among gram-negative bacteria (28), whereas NapR has a large molecular weight (27.6 kDa) and a low pI value (6.41 in *M. smegmatis* and 6.62 in *M. tuberculosis*). Thus, the physicochemical properties of NapR may differ from those of traditional NAPs, such as its stress response pathways, including its role in antioxidant defense.

Oxidative stress is an important and pervasive physical stress encountered by bacteria. ROS are generated as products of oxidative stress in bacteria. To counter oxidative stress, bacterial transcriptional regulators can sense the levels of ROS and coordinate an appropriate response. Nucleoid-associated proteins, important global regulatory factors, can help bacteria adapt to stress, such as oxidative stress. The nucleoid-associated protein Fis activates *topA* in response to oxidative stress in *E. coli* (29). WhiB4 dynamically manipulates both DNA architecture and gene expression to control the oxidative stress response in *Mycobacterium tuberculosis* (2). In this study, we identified a novel nucleoid-associated protein, NapR, that mediates bacterial antioxidant defense.

The antioxidant activity of the mycobacteria in this study was directly caused by the loss or reduced expression of *ggr*. The functions of the GGR in different species vary, but they are all related to the electron transport chain or redox reactions. In archaea, GGR catalyzes the formation of saturated isoprenoid chains, important components of their membrane lipids, which contribute to archaeal survival under extreme conditions (30). In mycobacteria, as a GGR family protein, MenJ catalyzes the reduction of a single double bond in the side chain of mycobacterial menaquinone isoprene, accompanied by the oxidative dehydrogenation of FADH_2_, resulting in a decrease in reducing power (21, 22). A previous study revealed that the survival rate of a *menJ* deletion mutant in macrophages greatly decreased (31), which also illustrates the importance of the GGR family in bacteria. On the basis of these findings, the reduction in reducing power in the *M. smegmatis ggr* deletion mutant may cause the electron transport chain to be blocked, resulting in a reduction in bacterial ROS levels, which eventually leads to H_2_O_2_ resistance in the mutant.

**Fig 8:**
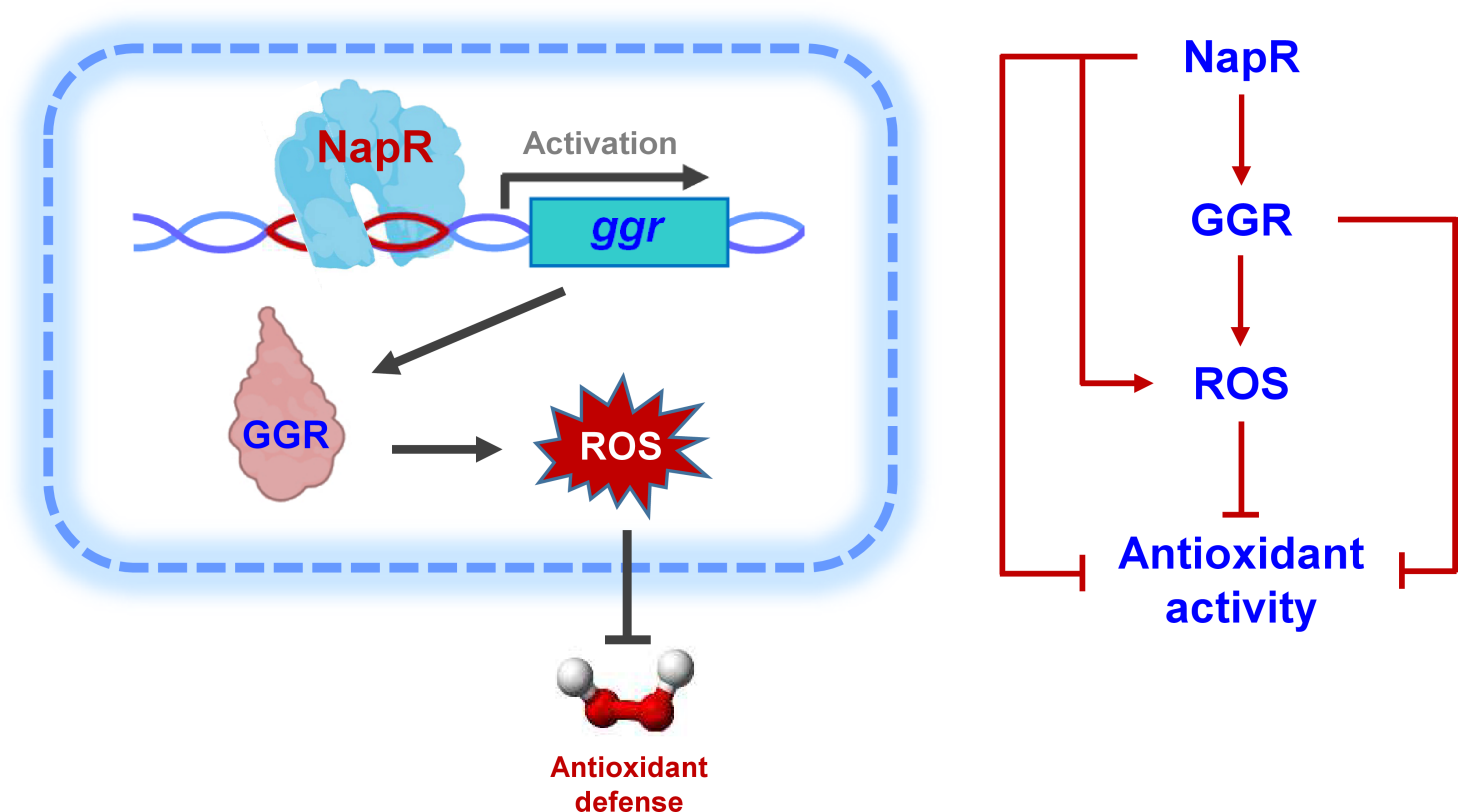
Regulatory model and pathway through which NapR regulates antioxidant defense in mycobacteria.

In this study, we report a novel nucleoid-associated protein, NapR, that positively regulates *ggr* to affect mycobacterial antioxidant defense. As shown in Fig. 8, NapR acts as an activator and positively regulates the expression of *ggr*, causing an increase in ROS in mycobacteria and ultimately decreasing bacterial antioxidant activity. In conclusion, our findings reveal a novel nucleoid-associated protein and provide new evidence for understanding antioxidant defense in bacteria.

## MATERIALS AND METHODS

### Expression and purification of proteins

The related genes were amplified by polymerase chain reaction (PCR) using their respective primer pairs (5′-ATGAGAATTCAAGTGCGGCGAGGGTCGAGATC-3′ and 5′-CGAGTCTAGACTAACTGGCGCGTTCGCGGC-3′ for *napR*_msm_, 5′-ATGAGAATTCAAGTGGTCAGAATCCCCCGACC-3′ and 5’-CGAGTCTAGATCAACGCACGGCGGGATTGT-3′ for *napR*_mtu_) from the genomic DNA of *M. smegmatis* mc^2^155 and *M. tuberculosis*. The amplified DNA fragments were subsequently purified and ligated into an engineered pET28a expression vector or pMV261 overexpression vector to construct recombinant plasmids. *E. coli* BL21 cells transformed with the recombinant plasmid were grown in 250 mL of LB medium. Protein expression was induced by 0.5 mM isopropyl β-D-1-thiogalactopyranoside. Afterward, the harvested cells were lysed, and the target protein was purified by affinity chromatography (32). Afterward, the purified protein was dialyzed for 3 h and stored at −80°C. Recombinant plasmids, such as pMV261-*napR*, and the corresponding recombinant strains were constructed.

### Determination of mycobacterial growth

The recombinant strains were grown to the mid-log phase in 7H9 medium supplemented with 30 µg/mL kanamycin. After they were harvested, the cells were suspended in fresh medium. Afterward, the cultures were added to 100 mL of fresh 7H9 medium with or without H_2_O_2_, ensuring that the initial concentrations were OD_600_ = 0.1. All the cultures were grown at 37°C in a shaker at 160 rpm. The samples were taken at different time points, and they were diluted and spread on 7H10 plates to count the number of colony-forming units. Three parallel experiments were performed for each sample. In addition, growth of all strains was monitored by measuring OD_600_ using the Agilent BioTek LogPhase 600 microbiology analyzer.

### β-Galactosidase activity assays

The fusion fragments of different promoters and the reporter gene *lacZ* were subsequently cloned and inserted into the pMV261 expression vector, after which β-galactosidase activity experiments were performed in *M. smegmatis*. The cloned recombinant plasmids were transformed into *napR*-deleted and wild-type strains to obtain the corresponding recombinant reporter strains. Enzyme activity assays were performed in different recombinant reporter strains (33).

### Electrophoretic mobility shift assay

The DNA fragments were generated through PCR amplification and purified using a DNA purification kit. DNA fragments were coincubated with various amounts of proteins in reaction buffer (50 mM Tris-HCl, 10 mM MgCl_2_, 1 mM dithiothreitol, and 50 mM NaCl) at room temperature for 15 min, and the total volume of the reaction mixtures was 20 µL. The mixtures were loaded onto 6% nondenaturing acrylamide gels and electrophoretically separated in 0.5× TBE buffer. Electrophoresis was performed at 150 V, and the resulting image was analyzed using a Bio-Rad GelDoc Go imaging system.

### Topoisomerase relaxation assay

The supercoiled pMind plasmid was used for the *in vitro* DNA relaxation assay. As previously described (34), the pMind plasmid was coincubated with various amounts of NapR at 37°C for 15 min, after which TopA (topoisomerase) was added to a mixture containing 100 mM Tris-HCl (pH 7.8), 5 mM EDTA, and 0.5% SDS. Finally, proteinase K was added to the system and reacted at 37°C for 30 min. The samples were identified through electrophoresis on ice on a 1.2% agarose gel at 80 V for 1.5 h.

### DNase I digestion assay

DNase I digestion assays were performed as previously described with slight modifications (35). In particular, 100 ng of linear DNA fragments containing 10 mM Tris-HCl (pH 8.0), 10 mM NaCl, and 0.5 mM EDTA were coincubated with different concentrations of NapR at 37°C for 15 min. Afterward, 1 U of DNase I was added to this mixture at 37°C for 15 min. The sample was then placed in a 75°C water bath for 10 min to terminate the reaction. Finally, proteinase K was added and reacted at 37°C for 30 min to degrade all the proteins. The samples were electrophoresed through 1% agarose gel at 80 V for 1 h.

### Colocalization assays for the NapR-green fluorescence protein fusion and chromosomal DNA

The gene fusions between the *napR* coding region and a sequence encoding GFP (eGFP) were produced, cloned, and inserted into the vector pMV261 to produce a recombinant vector, which was subsequently transformed into the host strain. DAPI staining solution was added to the culture medium of the host strain that grew to the logarithmic phase. The final concentration of DAPI was 1 µg/mL, and the culture was allowed to grow for 1 h. The cells were harvested, washed twice with ice-cold 0.5× PBS, and observed under a confocal fluorescence microscope (36).

### Atomic force microscopy assay

Free DNA fragments (600 bp) were used as the DNA substrate. Free DNA fragments were incubated with NapR in AFM buffer (10 mM HEPES [pH 7.5] and 5 mM NiSO₄) for 15 min at room temperature. A 10 µL aliquot of the mixture was absorbed and deposited onto a freshly cleaved mica surface. DNA without NapR was deposited under the same buffer conditions. After 2 min of incubation, the mica substrate was rinsed with ultrapure water, blotted with lint-free paper, and dried under an N_2_ stream at room temperature. Imaging was performed in tapping mode at a scan rate of 0.999 Hz with a resolution of 256 × 256 pixels using an AFM instrument (Dimension ICON, Bruker) (26, 37).

### ROS measurements

The levels of ROS were measured by the fluorescent dyes 2-dichlorodihydrofluorescein diacetate (H_2_DCFDA, MCE) (38) and dihydroethidium (DHE, Solarbio) (39). The strains were cultured to the mid-logarithmic phase. The cells were resuspended in PBS to obtain 5 × 10^8^ cells per milliliter for detection. Bacterial solution was added to 96-well bottom plates with 50 µM H_2_DCFDA or 50 µM DHE at 37°C for 1 h, and the fluorescence intensity of each well was read by a fluorescence microplate reader at Ex 480 nm/Em 520 nm (H_2_DCFDA) or at Ex 518 nm/Em 605 nm (DHE). The final fluorescence value of H_2_DCFDA represented the levels of ROS in different samples. The final fluorescence value of DHE represented the level of superoxide anions (O_2_^-^).

## Data Availability

All the data described are contained within the document.

## SUPPLEMENTAL MATERIAL

Supplemental figures msphere.00746-25-s0001.doc Fig. S1 to S5.

ASM does not own the copyrights to Supplemental Material that may be linked to, or accessed through, an article. The authors have granted ASM a non-exclusive, world-wide license to publish the Supplemental Material files. Please contact the corresponding author directly for reuse.
