# Supplementary material for: NapR, a novel nucleoid-associated protein, regulates antioxidant defense in mycobacteria
Source: mSphere. 2025 Nov 13;10(12):e00746-25. doi: 10.1128/msphere.00746-25 (PMC12724333; doi:10.1128/msphere.00746-25)
Supplement: Supplemental figures — Fig. S1 to S5. [file msphere.00746-25-s0001.doc]

**NapR, a novel** **nucleoid-associated** **protein, regulates** **antioxidant defense in mycobacteria**

**Kun Wang†, Xujie Cui†, Xiangyang Zhang, Jiachen Zheng, Xiaocui Ling, Yunfan Zhang, Pengbo Yu, Boyan Lv*, Weihui Li***

College of Life Science and Technology, Guangxi University, Guangxi Research Center for Microbial and Enzyme Engineering Technology, State Key Laboratory for Conservation and Utilization of Subtropical Agro-bioresources, Nanning 530004, China

**†**Co-first author: Kun Wang and Xujie Cui contributed equally to this work.

*To whom correspondence should be addressed:

College of Life Science and Technology, Guangxi University, Nanning 530004, China

Email: lwhlbx@163.com

Tel: +86-771-2852965

**A list of the supplementary materials:**

**Fig. S1.** NapR insertion mutation affects the antioxidant activity of *M. smegmatis*.

**Fig. S2.** NapR amino acid sequence alignment analysis in mycobacteria.

**Fig. S3.** NapR_mbb_ and chromatin DNA fluorescence colocalization assay.

**Fig.** **S4** Growth curve verification that NapR modulates the level of mycobacterial antioxidant defense through *ggr.*

**Fig. S5.** NapR regulates antioxidant activity by *ggr* in *M. smegmatis*.


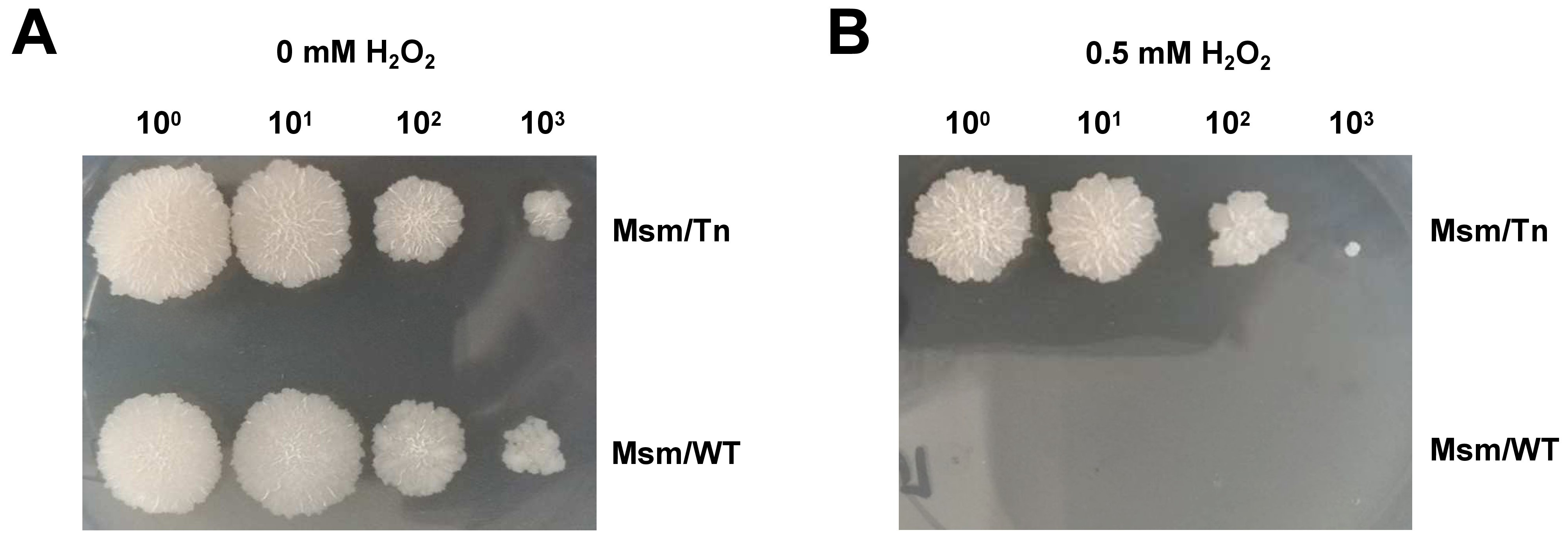


**Fig. S1.** **NapR insertion mutation affects the antioxidant activity of *M. smegmatis*.** **(A)** Middlebrook 7H10 without H_2_O_2_. **(B)** Middlebrook 7H10 with 0.5 mM H_2_O_2_. The top row is the screened *M. smegmatis* insertion mutant strain (Msm/Tn), and the bottom row is the *M. smegmatis* wild-type strain (Msm/WT). The dilution ratio of the bacterial solution was 1-fold (stock solution), 10-fold, 100-fold and 1000-fold from left to right.


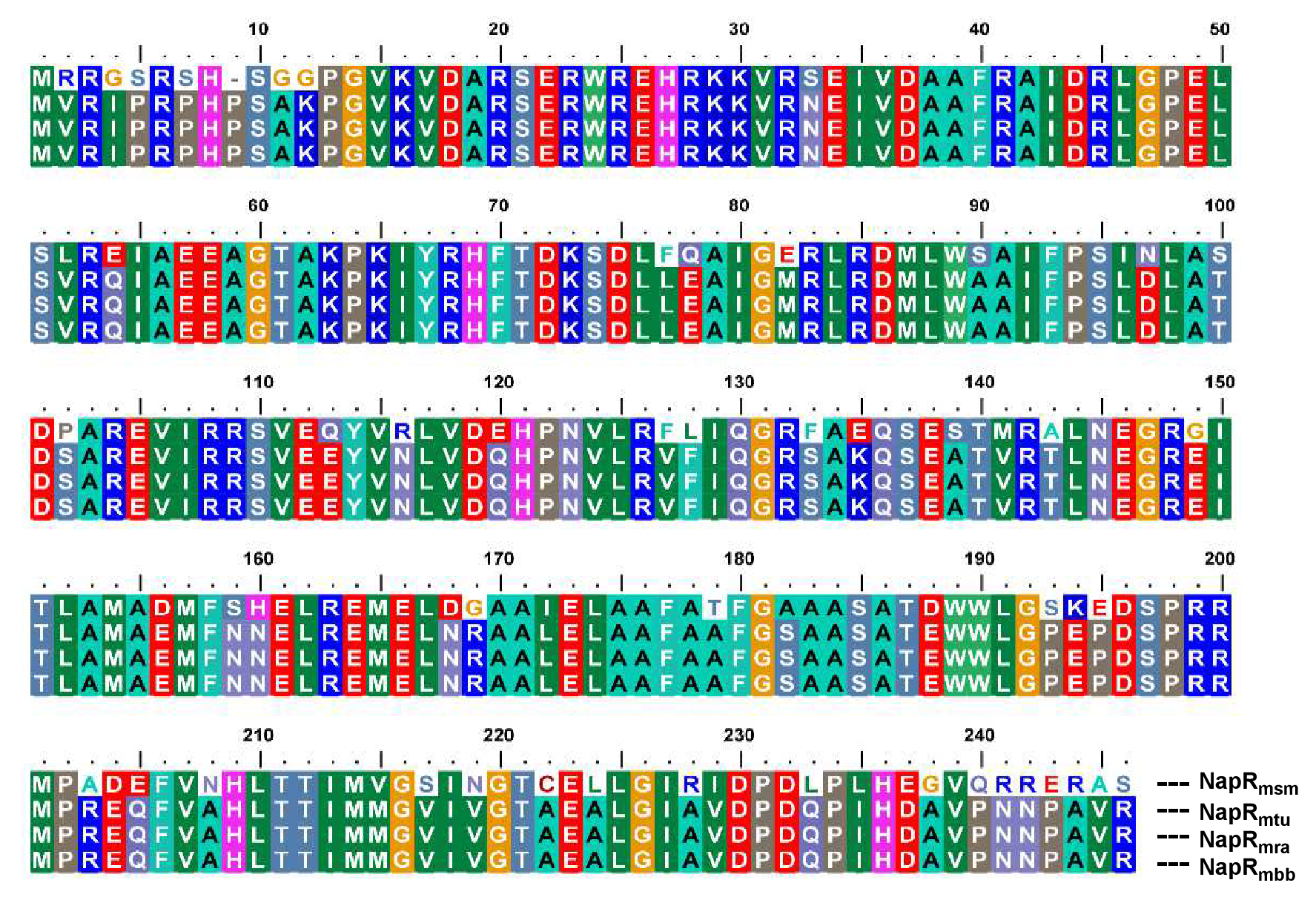


**Fig. S2. NapR amino acid sequence alignment analysis in mycobacteria, including *M. smegmatis*, *M. tuberculosis* H37Rv, *M. tuberculosis* H37Ra, and *M. bovis* BCG.**

**
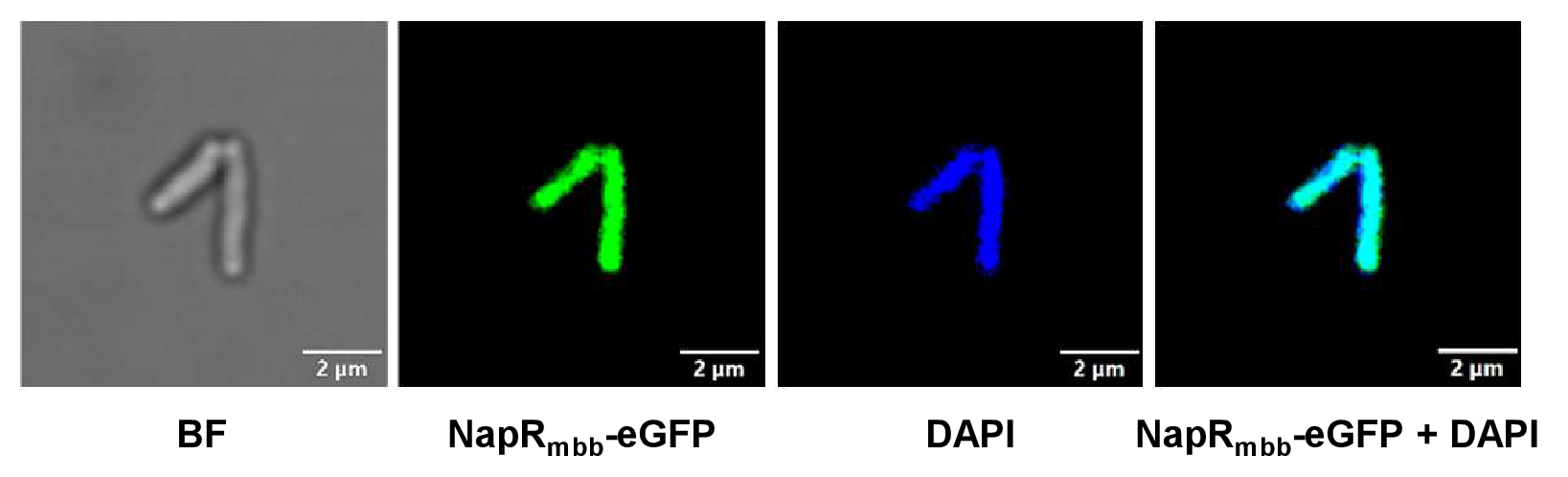
**

**Fig. S3. NapR_mbb_ and chromatin DNA fluorescence colocalization assay.** Colocalization assays for eGFP-tagged NapR_mbb_ with the nucleoid in *M. bovis* BCG. From left to right: BF, brightfield observation of BCG morphology; NapR_mbb_-eGFP, NapR_mbb_ was visualized by fusing with eGFP; DAPI, nucleoid was detected by staining with DAPI; NapR_mbb_-eGFP+DAPI, superimposition of eGFP-tagged NapR_mbb_ and DAPI-stained nucleoid. The scale bar in each figure is 2 µm.

**
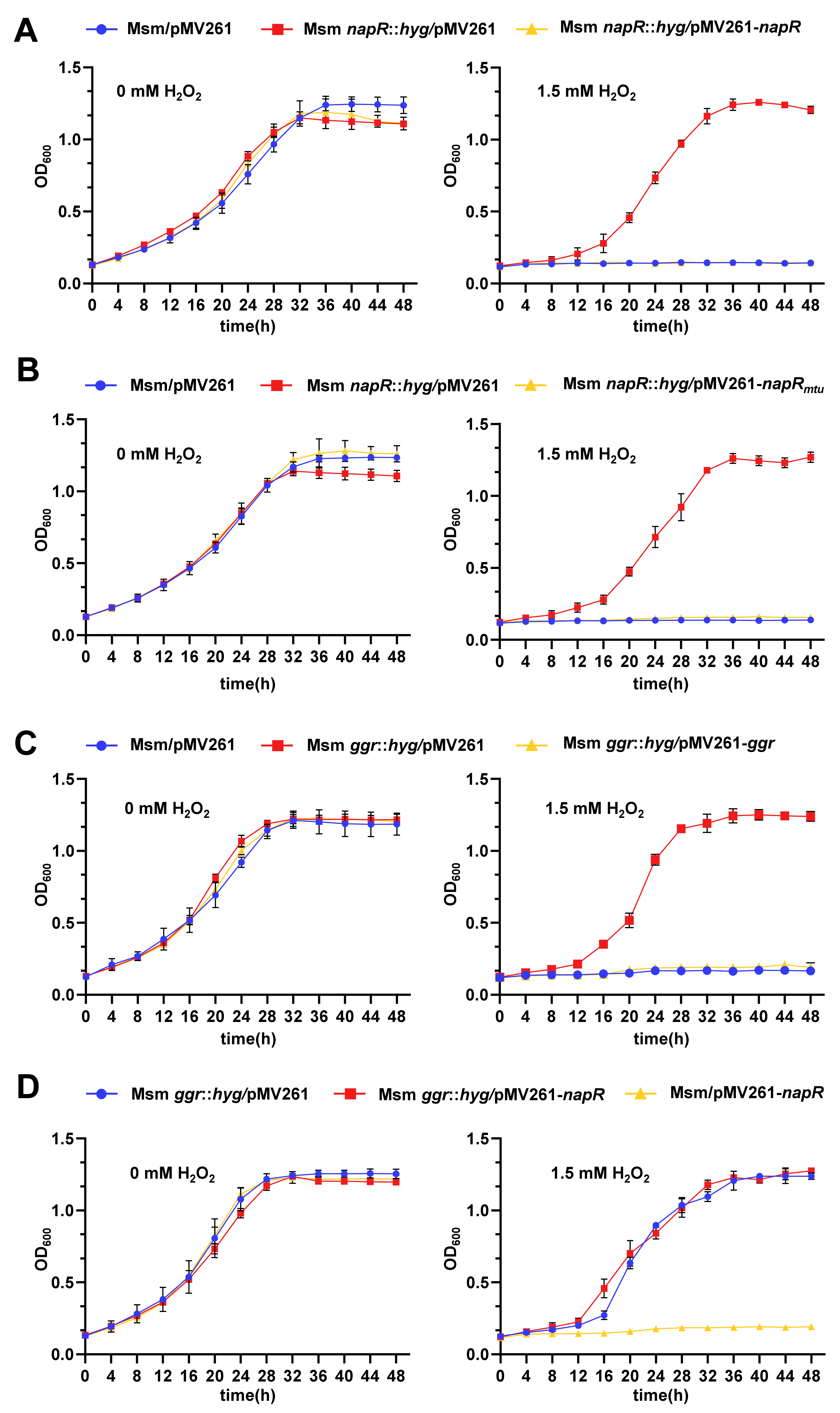
**

**Fig. S4. Growth curve verification that NapR modulates the level of mycobacterial antioxidant defense through *ggr*.** (A) Growth curves of the *M. smegmatis* wild-type strain (Msm/pMV261), *napR* deletion strain (Msm *napR*::*hyg*/pMV261) and complementary strain (Msm *napR*::*hyg*/pMV261-*napR*) with 0 mM H_2_O_2_ and 1.5 mM H_2_O_2_. (B) Growth curves of the *M. smegmatis* wild-type strain (Msm/pMV261), *napR*-deleted strain (Msm *napR*::*hyg*/pMV261), and cross-complementary strain (Msm *napR*::*hyg*/pMV261-*napR_mtu_*) with 0 mM H_2_O_2_ and 1.5 mM H_2_O_2_. (C) Growth curves of the *M. smegmatis* wild-type strain (Msm/pMV261), *ggr*-deleted strain (Msm *ggr*::*hyg*/pMV261) and complementary strain (Msm *ggr*::*hyg*/pMV261-*ggr*) with 0 mM H_2_O_2_ and 1.5 mM H_2_O_2_. (D) Growth curves of *ggr*-deleted strain (Msm *ggr*::*hyg*/pMV261), cross-recombinant strain (Msm *ggr*::*hyg*/pMV261-*napR*) and the *napR*-overexpressing strain (Msm/pMV261-*napR*), with 0 mM H_2_O_2_ and 1.5 mM H_2_O_2_.

**
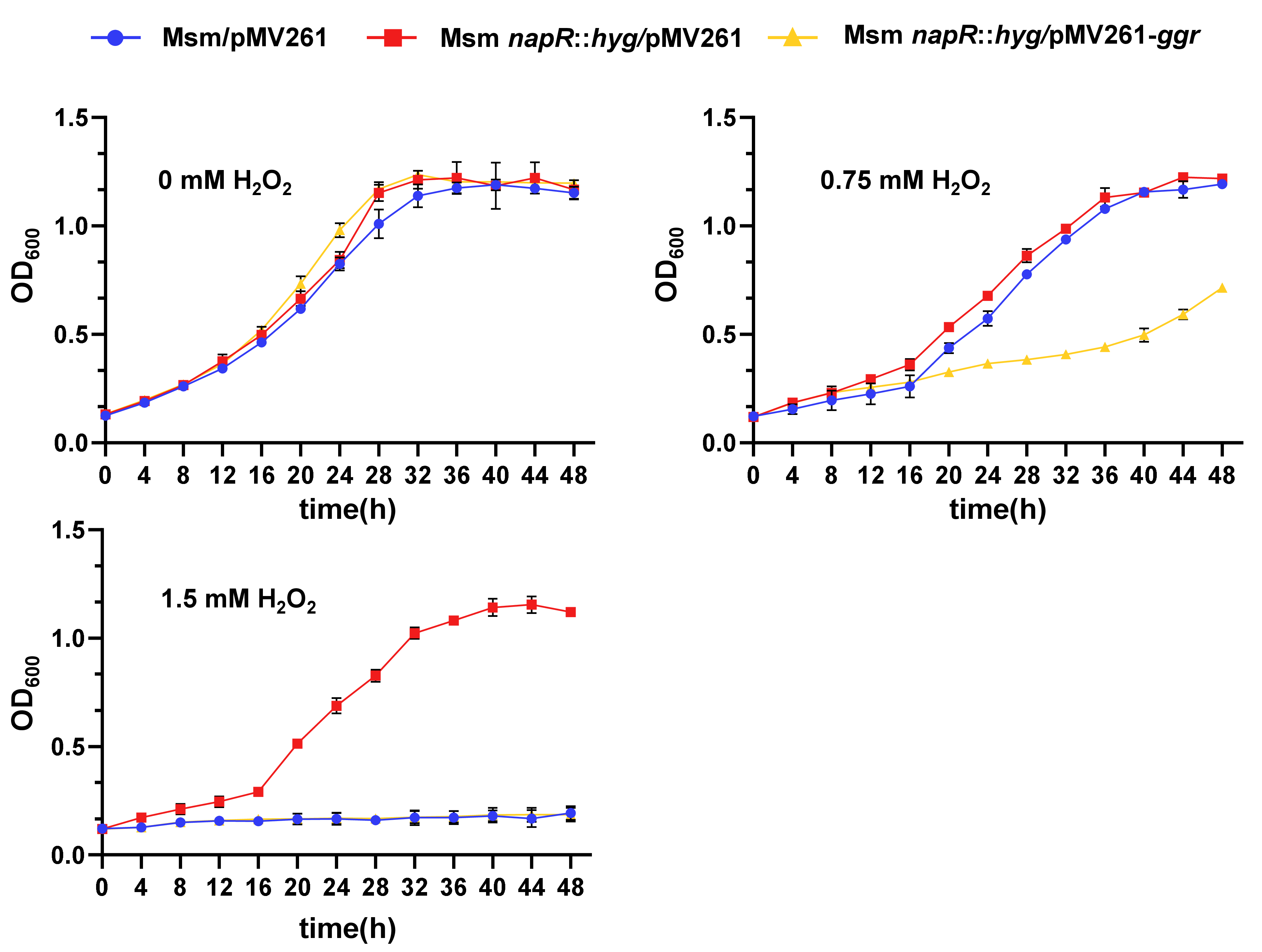
**

**Fig. S5.** **NapR regulates antioxidant activity by *ggr* in *M. smegmatis*.** Growth curve of the strain (Msm/pMV261), *napR* deleted strain (Msm *napR*::*hyg*/pMV261) and cross-recombinant strain (Msm *napR*::*hyg*/pMV261-*ggr*) with 0 mM H_2_O_2_, 0.75 mM H_2_O_2_ and 1.5 mM H_2_O_2_.
